# Antiallergic Phorbol Ester from the Seeds of *Aquilaria malaccensis*

**DOI:** 10.3390/ijms17030398

**Published:** 2016-03-21

**Authors:** Michal Korinek, Vitthal D. Wagh, I-Wen Lo, Yu-Ming Hsu, Hsue-Yin Hsu, Tsong-Long Hwang, Yang-Chang Wu, Yuan-Bin Cheng, Bing-Hung Chen, Fang-Rong Chang

**Affiliations:** 1 Graduate Institute of Natural Products, College of Pharmacy, Kaohsiung Medical University, Kaohsiung 807, Taiwan; mickorinek@hotmail.com (M.K.); vdwagh22@gmail.com (V.D.W.); iwenlo99@gmail.com (I.-W.L.); u98531010@kmu.edu.tw (Y.-M.H.); yachwu@mail.cmu.edu.tw (Y.-C.W.); jmb@kmu.edu.tw (Y.-B.C.); 2 Department of Biotechnology, College of Life Science, Kaohsiung Medical University, Kaohsiung 807, Taiwan; 3 Department of Life Sciences, Tzu Chi University, Hualien 970, Taiwan; hsueyin@mail.tcu.edu.tw; 4 Graduate Institute of Natural Products, College of Medicine, Chang Gung University, Taoyuan 333, Taiwan; htl@mail.cgu.edu.tw; 5 Research Center for Industry of Human Ecology and Graduate Institute of Health Industry Technology, Chang Gung University of Science and Technology, Taoyuan 333, Taiwan; 6 Department of Anesthesiology, Chang Gung Memorial Hospital, Taoyuan 333, Taiwan; 7 School of Pharmacy, College of Pharmacy, China Medical University, Taichung 404, Taiwan; 8 Chinese Medicine Research and Development Center, China Medical University Hospital, Taichung 404, Taiwan; 9 Center for Molecular Medicine, China Medical University Hospital, Taichung 404, Taiwan; 10 Center for Infectious Disease and Cancer Research, Kaohsiung Medical University, Kaohsiung 807, Taiwan; 11 The Institute of Biomedical Sciences, National Sun Yat-Sen University, Kaohsiung 804, Taiwan; 12 Cancer Center, Kaohsiung Medical University Hospital, Kaohsiung 807, Taiwan; 13 Department of Marine Biotechnology and Resources, National Sun Yat-sen University, Kaohsiung 804, Taiwan

**Keywords:** *Aquilaria malaccensis* seeds, antiallergic, degranulation, phorbol ester, bioactivity-guided fractionation

## Abstract

The *Aquilaria malaccensis* (Thymelaeaceae) tree is a source of precious fragrant resin, called agarwood, which is widely used in traditional medicines in East Asia against diseases such as asthma. In our continuous search for active natural products, *A. malaccensis* seeds ethanolic extract demonstrated antiallergic effect with an IC_50_ value less than 1 µg/mL. Therefore, the present research aimed to purify and identify the antiallergic principle of *A. malaccensis* through a bioactivity-guided fractionation approach. We found that phorbol ester-rich fraction was responsible for the antiallergic activity of *A. malaccensis* seeds. One new active phorbol ester, 12-*O*-(2*Z*,4*E*,6*E*)-tetradeca-2,4,6-trienoylphorbol-13-acetate, aquimavitalin (**1**) was isolated. The structure of **1** was assigned by means of 1D and 2D NMR data and high-resolution mass spectrometry (HR-MS). Aquimavitalin (**1**) showed strong inhibitory activity in A23187- and antigen-induced degranulation assay with IC_50_ values of 1.7 and 11 nM, respectively, with a therapeutic index up to 71,000. The antiallergic activities of *A. malaccensis* seeds and aquimavitalin (**1**) have never been revealed before. The results indicated that *A. malaccensis* seeds and the pure compound have the potential for use in the treatment of allergy.

## 1. Introduction

Imunoglobulin E (IgE)-mediated allergy is a common immune system disorder affecting approximately 235 million people worldwide, particularly the population in developed countries [1]. Although today we are able to treat the symptoms of allergy, available medications have undesirable effects, especially with a prolonged use. Therefore, there is a need to search for alternative treatment. In general, some natural sources are considered as safe and easily available. Mast cells and their degranulation play a crucial role in IgE-mediated allergic inflammatory responses, such as allergic rhinitis, acute asthma, and atopic eczema [2]. β-Hexosaminidase is an enzyme released along with histamine from mast cells (rat basophilic leukemia cells, RBL-2H3 cells) upon activation and serves as a well-accepted *in vitro* model in allergy [3].

Agarwood is a priceless fragrant resinous wood from the *Aquilaria* species (*Thymelaeaceae)*, which is formed as a defense mechanism to fend off pathogens. Agarwood is widely used in religious, aromatic, and medicinal preparations [4,5]. *Aquilaria* species has been traditionally used in Thai [6] and Korean [7] medicine, in the Ayurvedic practice, as well as traditional Chinese medicine to treat various diseases, particularly the diseases associated with inflammation [8]. Agarwood from the *Aquillaria* species has been used as cardiotonic, carminative, antiasthmatic, aphrodisiac, astringent remedy, and has been found effective against diarrhea, dysentery, gout, rheumatism, paralysis, and parasites, and it has been beneficial for skin diseases [9]. The *Aquilaria* species was previously found to possess antidepressant [10,11], antineuroinflammatory [12], analgesic, antiinflammatory [13], antioxidant, antibacterial [6], antihyperglycemic *in vivo* [14], and laxative activity *in vivo* [15].

*Aquilaria malaccensis* Lam. (syn. *Aquilaria agallocha* Roxb.) (Thymelaeaceae) is a tropical tree native to Malaysia, locally known as “Karas”. It is distributed in the rainforests of Indonesia, Thailand, Cambodia, Laos, Malaysia, Philippines, and India [16]. The alcoholic extract of *A. malaccensis* stems and bark exhibited cardiotonic activity [17], and cytotoxicity against Eagle’s carcinoma of the nasopharynx and P-388 lymphocytic leukemia cells *in vitro* [18]. The aqueous extract showed antitrypanosomal [19], antibacterial [20], and antiallergic activity *in vitro* and *in vivo* [7]. The study on the composition of agarwood from *A. malaccensis* utilizing gas chromatography-mass spectrometry (GC-MS) revealed the presence of chromones, aromatic compounds, sesquiterpenes, monoterpenes, steroids and fatty acids [21]. In a previous phytochemical investigation, feruryl glyceride and phorbol ester were isolated from *A. malaccensis* bark [18].

However, there was no investigation reporting on composition and bioactivity of *A. malaccensis* seeds (AMS).

In the current study, we investigated antiallergic, antiinflammatory, and cytotoxic activities of AMS extract and its fractions. Within a project of continuous screening for active natural products, AMS showed strong antiallergic effect with an IC_50_ value less than 1 µg/mL in degranulation assay. Therefore, a phytochemical investigation of AMS was undertaken through a bioactivity-guided fractionation approach. The active components of the most active fraction were further defined as a mixture of phorbol esters, and, moreover, the new active phorbol ester possessing polyunsaturated fatty acid (**1**) was isolated.

## 2. Results and Discussion

### 2.1. Antiallergic, Antiinflammatory, Cytotoxic Effects of A. malaccensis Seeds (AMS)

The preliminary bioactivity screening of AMS ethanolic extract (A-EtOH) showed potent antiallergic (IC_50_ 0.92 and 3.9 µg/mL in A23187 and antigen-induced β-hexosaminidase assay, respectively) (Table 1), and antiinflammatory activities (90.1% and 85.3% inhibition of superoxide generation and elastase release at 10 µg/mL, respectively) (Table 2). All partitioned fractions except water layer displayed significant antiallergic and antiinflammatory activities (A-BuOH, A-EtOAc, A-Hexane, A-MeOH).

The effects of the AMS samples on degranulation in both A23187- and antigen-induced β-hexosaminidase assays were dose-dependent (Tables S1 and S2). To clarify that antiallergic activity of the samples was due to inhibition of β-hexosaminidase release, and not false positive as a result of direct inhibition of β-hexosaminidase enzymatic activity [22], the enzyme was extracted and tested with the active samples. None of the samples inhibited the enzymatic activity of β-hexosaminidase (Table 1).

As the methanol layer proved the best antiallergic activity (IC_50_ 0.0089 and 0.069 µg/mL in A23187 and antigen-induced degranulation assay, respectively), it was further separated using silica gel column chromatography to yield six fractions, AM1–AM6 (subfractions of methanol layer from *Aquilaria malaccensis* seeds). Among them, fraction AM4 showed the most remarkable antiallergic activity inhibiting β-hexosaminidase release from mast cells induced by either A23187 (IC_50_ 0.0034 µg/mL) or antigen (IC_50_ value 0.0065 µg/mL).

**Figure 1:**
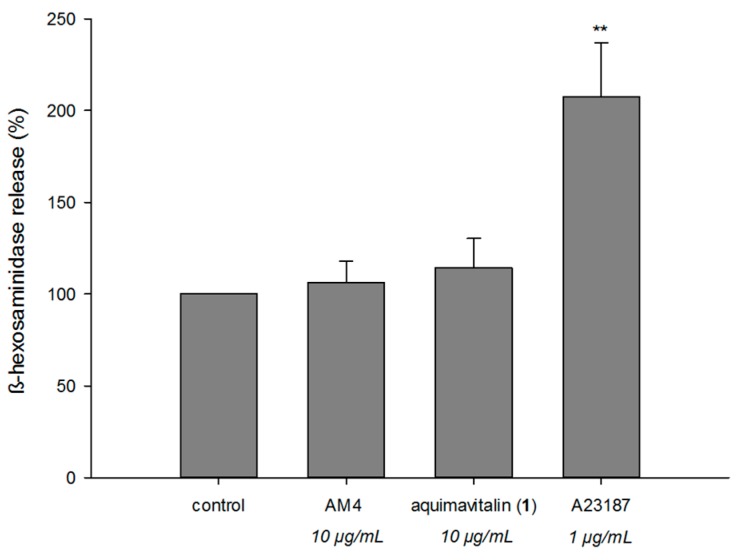
Activity of phorbol ester-rich fraction (AM4) and aquimavitalin (**1**) on stimulant-free degranulation in RBL-2H3 cells. The RBL-2H3 cells were treated with AM4 (10 µg/mL) and aquimavitalin (10 µg/mL) for 10 h. Tyrode’s buffer supplemented with glucose, bovine serum albumin (BSA) and glutamine was used as a medium. A23187 (1 µM) was used as a positive control. Data are expressed as mean ± SD (n = 3). ** *p* < 0.001 compared with the control value.

In cytotoxicity assay against a panel of three cancer cell lines (human hepatocellular carcinoma cells HepG2, human breast adenocarcinoma cells A549, and human lung adenocarcinoma cells MDA-MB231), only some of the AMS fractions showed cytotoxic activities at a 20-µg/mL level (Table 3) (A-BuOH 57.1% against A549, AM4 56.5% against MDA-MB231 and 79.3% against A549, AM6 56.0% against MDA-MB231 cell line). Moreover, considering weak cytotoxicity of AMS towards RBL-2H3 cells, the antiallergic active fraction AM4 exerted therapeutic index up to 28,000. To further rule out the possibility that AM4 causes direct mast cell activation, we examined the capacity of AM4 to elicit degranulation by itself. Results showed that the AM4 treatments did not cause significant degranulation as compared with untreated control (Figure 1). These data implied that AM4 is the best target for further phytochemical analysis.

### 2.2. Chemical Analysis and Bioactivity-Guided Fractionation

Following bioactivity-guided fractionation of the active fractions, the AM4 was further separated, yielding several active fractions, AM4-3, AM4-4, and AM4-5 (Tables S1 and S2).

**Figure 2:**
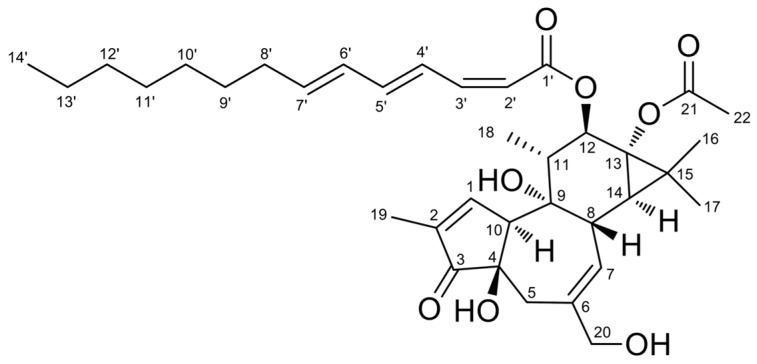
Structure of aquimavitalin (**1**).

AM4-4 (IC_50_ 4.8 × 10^−5^ µg/mL, therapeutic index 1477328, A23187-induced; and IC_50_ 6.8 × 10^−4^ µg/mL, therapeutic index 103776, antigen-induced β-hexosaminidase assay) afforded the most active fraction AM4-4-8 (IC_50_ 7.6 × 10^−6^ µg/mL, therapeutic index 9645374, A23187-induced; and IC_50_ 8.0 × 10^−5^ µg/mL, therapeutic index 917440, antigen-induced degranulation assay), and a new compound, aquimavitalin (**1**) (IC_50_ values of 0.0017 µM, therapeutic index 71,538, A23187-induced; and IC_50_ 0.011 µM, therapeutic index 10,550, antigen-induced degranulation assay) (Figure 2).

According to ^1^H NMR of the crude (A-EtOH), methanolic (A-MeOH) and the subsequent active fractions (AM4 and AM4-4) (Figure S1), we found the proportional relationship of the antiallergic activity with the increase in signals typical for phorbol diterpenes (*δ*_H_ 7.5, H-1; *δ*_H_ 5.6, H-7; *δ*_H_ 4.0, H-20).

### 2.3. Structure Elucidation of Aquimavitalin (**1**)

Compound **1** (Figures S2–S8) was isolated as a colorless oil. It was assigned the molecular formula C_36_H_50_O_8_Na, according to high-resolution electrospray ionization mass spectrometry (HR-ESIMS) (*m*/*z* 633.33980 [M + Na]^+^, calcd. 633.33979), indicating 12 degrees of unsaturation. Its IR spectrum revealed the presence of hydroxyl (3413 cm^−1^), carbonyl (1710 cm^−1^) and olefinic (1615 cm^−1^) functionalities.

The NMR data of compound **1** (^1^H, ^13^C and heteronuclear multiple quantum coherence, HMQC, Table 4) confirmed the presence of α, β-unsaturated carbonyl (*δ*_H_ 7.57, s, H-1, *δ*_C_ 160.8, C-1; *δ*_C_ 132.8, C-2; *δ*_C_ 209.3, C-3), trisubstituted double bond (*δ*_H_ 5.68, brs, H-7, *δ*_C_ 129.1, C-7; *δ*_C_ 140.6, C-6), oxygenated methylene (*δ*_H_ 3.95, d, *J* = 12.8 Hz, 4.02, d, *J* = 12.8 Hz, H-20, *δ*_C_ 67.9, C-20), oxygenated methane (*δ*_H_ 5.43, d, *J* = 10.4 Hz, H-12, *δ*_C_ 75.9, C-12), four methyls, a methylene and four methines. Furthermore, signals for acetyl group (*δ*_H_ 2.10, s, H-22, *δ*_C_ 21.1, C-22; *δ*_C_ 173.9, C-21) and fatty acid moiety including six olefinic protons, six methylenes and terminal methyl group were detected. The ^1^H NMR data was closely related to known compound 12-*O*-(2*Z*,4*E*,6*E*)-deca-2,4,6-trienoylphorbol-13-acetate [18] except of the length of the fatty acid moiety (Table S3).

**Figure 3:**
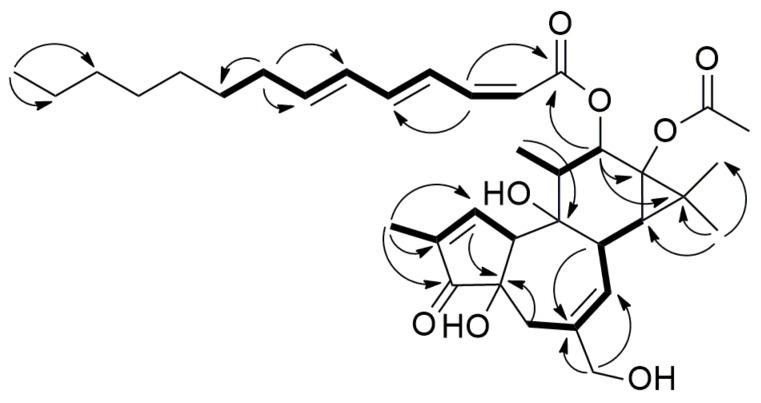
Key ^1^H–^1^H correlation spectroscopy (COSY) (bold) and HMBC (arrow) correlations of aquimavitalin (**1**).

The ^1^H–^1^H correlation spectroscopy (COSY) correlations (Figure 3) indicated the presence of C-10/C-1/C-19, C-5/C-7/C-8/C-14, and C-12/C-11/C-18 moieties for backbone, C-2’/C-3’/C-4’/C-5’/C-6’/C-7’/C-8’/C-9’ for fatty acid moiety. The COSY correlations together with long-range heteronuclear multiple bond correlation spectroscopy (HMBC) correlations (Figure 3) from H-19/C-1, C-2, C-3; H-1/C-4; H-5/C-4; H-20/C-5, C-6, C-7; H-8/C-6, C-14, C-15; H-12 to C-11, C-13, C-15, C-18; H-18/C-9; H-16 and H-17/ C-13, C-14, C-15 established the tigliane (phorbol) type diterpene backbone of compound **1** [23,24].

**Figure 4:**
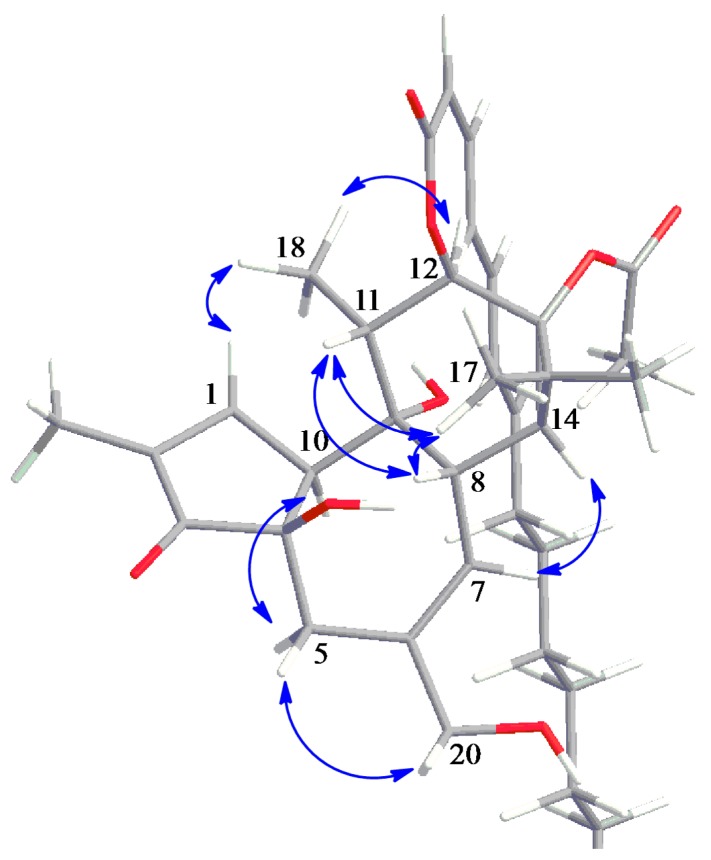
Key NOESY (double-headed arrow) correlations of aquimavitalin (**1**). Grey color represents carbon, red color oxygen and white color proton.

The relative configuration was assigned by means of nuclear Overhauser effect spectroscopy (NOESY) correlations of **1** (Figure 4). The cross-peaks of H-8/H-11, H-11/H-17 and H-17/H-8 indicated that they are all β-oriented. Moreover, the correlation between H-1/H18/H-12 suggested that the fatty acid moiety is also β-oriented [23]. Additionally, **1** showed negative specific optical rotation (−3.8) similar to 12-*O*-(2*Z*,4*E*,6*E*)-deca-2,4,6-trienoylphorbol-13-acetate (−15.3) [18].

The fatty acid was identified as (2*Z*,4*E*,6*E*)-tetradeca-2,4,6-trienoic acid according to 1D NMR and COSY correlations supported by following HMBC correlations, H-3′/C-1′ (*δ*_C_ 166.3), C-5′ (*δ*_C_ 142.4); H-8′/C-6′ (*δ*_C_ 130.1), C-7′ (*δ*_C_ 141.0), C-9′ (*δ*_C_ 28.9); H-9′/C-10′ (*δ*_C_ 29.1) and H-14′/C-12′ (*δ*_C_ 31.7), C-13′ (*δ*_C_ 22.6). The geometry of the double bonds was assigned by coupling constants in ^1^H NMR. The NMR data were in agreement with those of (2*Z*,4*E*,6*E*)-ethyl tetradeca-2,4,6-trienoate [25]. The fatty acid moiety was attached to phorbol backbone at C-12 by virtue of HMBC correlation from H-12 to C-1′ (*δ*_C_ 166.3). Therefore, compound **1** was identified as 12-*O*-(2*Z*,4*E*,6*E*)-tetradeca-2,4,6-trienoylphorbol-13-acetate and named as aquimavitalin.

### 2.4. Antiallergic Activity of Aquimavitalin (**1**)

In degranulation assay, aquimavitalin (**1**) showed significant β-hexosaminidase release-inhibitory activity with IC_50_ values of 0.0017 µM (therapeutic index 71,538) using A23187 as an inducer and 0.011 µM (therapeutic index 10,550) using antigen as an inducer. Aquimavitalin (**1**) did not inhibit β-hexosaminidase enzymatic activity (Table 1), neither trigger the degranulation of unstimulated mast cells (Figure 1). According to our results, phorbol ester-rich fractions (AM4-4, AM4-4-8) showed stronger activity (up to pg/mL level) than a pure compound. This phenomenon may be a result of synergistic effects of phorbol esters in the mixture.

In general, phorbol esters, particularly phorbol-12-myristate-13-acetate (PMA), are well-known as irritant, proinflammatory and cocarcinogenic. Nevertheless, phorbol esters were previously reported to exert antiinflammatory, anti-HIV, antiparasitic and anticancer activities [26]. Both free C-20 hydroxy, and C-12 and/or C-13 ester moieties were important for the activities of phorbol esters [26]. Importantly, it was suggested that unsaturation of ester functionality may play a crucial role in bioactivity of phorbols [26,27]. Previously, 12-*O*-(2*Z*,4*E*,6*E*)-deca-2,4,6-trienoylphorbol-13-acetate, a phorbol ester possessing similar conjugated fatty acid moiety as **1**, was isolated from *A. malaccensis* bark and exerted cytotoxic activity in P-388 lymphocytic leukemia cells *in vitro* [18]. In structure-activity relationship study on phorbol esters containing fatty acids with different level of unsaturation and carbon chain length, phorbol esters carrying conjugated unsaturated fatty acid as acyl group showed irritant but very weak tumor-promoting activities. [27]. This is the first study to report on the antiallergic potential of pure phorbol ester with the therapeutic index up to 71,000. The antiallergic activity of AMS together with identification of its active component provides scientific support for the folk use of *A. malaccensis* against asthma.

## 3. Materials and Methods

### 3.1. General Procedures

Sephadex LH-20 (Merck KGaA, Darmstadt, Germany), silica gel 60 (Merck KGaA) and Geduran Si 60 (Merck KGaA) were used for column chromatography. TLC plates (Silica Kiesel 60 F254) were from Merck KGaA. Jasco V-530 ultraviolet spectrophotometer (Jasco International Co., Ltd, Tokyo, Japan) was used to measure UV spectra. IR spectra were obtained on an FT-IR-4100 Jasco spectrophotometer (Jasco). Optical rotations were achieved by a Jasco P-2000 digital polarimeter (Jasco). NMR spectra were obtained by JEOL JNM ECS 400 MHz. Electrospray ionization mass spectrometry (ESIMS) data were collected on a Waters micromass ZQ mass spectrometer (Waters Corporation, Milford, MA, USA). High-resolution ESIMS data was accomplished by a Bruker APEX II spectrometer (FT-ICR/MS, FTMS) (Bruker Daltonics Inc., Billerica, MA, USA). Dulbecco’s modified Eagle’s medium (high glucose) powder (DMEM), 3-(4,5-dimethylthiazol-2-yl)-2,5-diphenyltetrazolium bromide (MTT), *p*-nitrophenyl-*N*-acetyl-d-glucosaminide (*p*-NAG), penicillin and streptomycin, dexamethasone, calcium ionophore A23187, and dimethyl sulfoxide (DMSO) were purchased from Sigma-Aldrich (St. Louis, MO, USA). Fetal bovine serum (FBS) was obtained from Hyclone (Logan, UT, USA). Mouse anti-DNP IgE antibody was a generous gift from Dr. Daniel H. Conrad (Virginia Commonwealth University, Richmond, VA, USA).

### 3.2. Plant Material

The seeds of *A. malaccensis* were obtained from Hsue-Yin Hsu, Tzu Chi University, Hualien, Taiwan, in November 2014. The plant material was identified by Hsue-Yin Hsu, Department of Life Sciences, Tzu Chi University, Hualien, Taiwan. A voucher specimen (code no. KMU-AMS 1) was deposited in the Graduate Institute of Natural Products, College of Pharmacy, Kaohsiung Medical University, Kaohsiung, Taiwan.

### 3.3. Extraction and Isolation

Air-dried and powdered seeds of *A. malaccensis* (462 g) were extracted with 90% EtOH at room temperature (3 × 5 L) and then concentrated under reduced pressure. The combined extracts were concentrated and obtained crude ethanolic extract (A-EtOH, 27.7 g) was suspended in water and partitioned with ethyl acetate (3 × 1 L). The water layer was partitioned with *n*-butanol (3 × 1 L) to yield water layer (A-Water, 1654.0 g) and *n*-butanol layer (A-BuOH, 398.2 g). The EtOAc layer (A-EtOAc, 25.6 g) was further partitioned with *n*-hexane and 90% aqueous MeOH to obtain *n*-hexane layer (A-Hexane, 7.1 g) and MeOH layer (A-MeOH, 16.2 g). The MeOH layer (A-MeOH) was subjected to a column chromatography over silica gel (23 cm × 4 cm, silica gel 60, 0.063–0.200 mm, Merck) under a gradient elution of *n*-hexane/CH_2_Cl_2_/MeOH to yield six fractions (AM1, 6:3:1; AM2, 6:4:1; AM3, 6:6:1; AM4, 6:8:1; AM5, 6:10:1 and AM6, 6:10:2). Following bioactivity data, fraction AM4 (3212.0 g) was further fractionated over a Sephadex LH-20 column (CH_2_Cl_2_/MeOH, 1:1) to obtain eight sub-fractions (AM4-1 to AM4-8). Fraction AM4-3 (762.0 mg) was subjected to column chramtography (17 cm × 4 cm, Geduran Si 60, 0.040-0.063 mm, Merck) under gradient elution of EtOAc/*n*-hexane (from 1:10 to 4:1) yielding 15 fractions. Fraction AM4-4 (173.7 mg) was further separated by column chromatography on silica gel (30 cm × 1.5 cm, Geduran Si 60, 0.040–0.063 mm, Merck) under gradient elution of EtOAc/*n*-hexane (from 1:15 to 4:1) to obtain fraction AM4-4-7 (37.6 mg) and AM4-4-8 (6.8 mg) and aquimavitalin (**1**) (43.9 mg) together with other 8 subfractions. The yield of aquimavitalin (**1**) was 0.0095% from dry plant material, 0.16% from crude EtOH extract.

### 3.4. Experimental Data of Aquimavitalin (**1**)

Aquimavitalin (**1**): Colourless oil; ${\left[\alpha \right]}_{D}^{25}$ −3.75 (c 0.067, CHCl_3_); UV (MeOH) λ_max_ (log ε) 303 (2.78), 233 (2.75) nm; IR (neat) *v*_max_ 3413, 2965, 2922, 1710, 1615, 1377, 1258, 1092, 802; ^1^H NMR (CDCl_3_, 400 MHz) and ^13^C NMR (CDCl_3_, 100 MHz): see Table 4; ESIMS found m/z 611.3 [M + H]^+^ and 633.3 [M + Na]^+^; HR-ESIMS found (m/z 633.33980 [M + Na]^+^, (calcd. for C_36_H_50_O_8_Na: 633.33979).

### 3.5. Cell Culture

The mucosal mast cell-derived rat basophilic leukemia (RBL-2H3) cell line was purchased from the Bioresource Collection and Research Center (Hsin-Chu, Taiwan). Cells were grown in DMEM medium supplemented with 10% FBS and 100 U/mL penicillin plus 100 μg/mL streptomycin. Cells were cultured in 10 cm cell culture dishes at 37 °C in a humidified chamber with 5% CO_2_ in air.

### 3.6. Cell Viability Assay

A methylthiazol tetrazolium (MTT) assay was used to measure the potential toxic effects of the samples on RBL-2H3 cells [28]. Briefly, RBL-2H3 cells (2 × 10^4^ cells/well) were seeded in a 96-well plate overnight and treated with various concentrations of samples (10–100 μg/mL) for 24 h. MTT solution (0.5 mg/mL) was added to the wells (80 μL per well) and incubated for 1 h. The formed formazan crystals were dissolved in DMSO (80 μL). The absorbance at 595 nm was measured using microplate reader (Multiskan Ascent, Thermo Scientific, Waltham, MA, USA). The degree of cell viability of each sample was calculated as the percentage of control value (untreated cells). The maximal tolerated dose of DMSO was 0.5%. All experiments were repeated at least two times.

### 3.7. Degranulation β-Hexosaminidase Assay Induced by A23187 or Antigen

The degree of A23187- and antigen-induced degranulation in RBL-2H3 cells was determined by a β-hexosaminidase release assay as described previously [28,29] with following modifications. RBL-2H3 cells were seeded in a 96-well plate (2 × 10^4^ cells/well) for A23187-induced and in 48-well plate (3 × 10^4^ cells/well) for antigen-induced experiment. Cells were treated with various concentrations of the samples for 20 h. Dexamethasone (10 nM) was used as a positive control. The cells for the antigen-induced experiment were first sensitized with anti-DNP IgE (5 μg/mL) for at least 2 h. After thorough washing by pre-warmed Tyrode’s buffer (135 mM NaCl, 5 mM KCl, 1.8 mM CaCl_2_, 1.0 mM MgCl_2_, 5.6 mM glucose, 20 mM HEPES at pH 7.4), the cells were stimulated by either calcium ionophore A23187 (1 μM) or antigen DNP-BSA (100 ng/mL) in Tyrode’s buffer for 1 h. Unstimulated cells were either lysed with 0.5% Triton X-100 solution for the total amount of β-hexosaminidase release or left untreated for spontaneous release of β-hexosaminidase. Then aliquots of supernatants (50 μL) were incubated with equal volume of 1 μM of p-NAG (50 μL) prepared in 0.1 M citrate buffer (pH 4.5) serving as a substrate for the released β-hexosaminidase. After 1 h of incubation at 37 °C, the reaction was quenched by the addition of 100 μL of stop buffer (0.1 M Na_2_/NaHCO_3_, pH 10.0). Absorbance was measured at 405 nm on a microplate reader (Multiskan Ascent, Thermo Scientific). The inhibition percentage of β-hexosaminidase release was calculated as the percentage of control value (untreated stimulated cells). The maximal tolerated dose of DMSO was 0.5%. All experiments were repeated three times.

### 3.8. Effect on Enzymatic Activity of β-Hexosaminidase

To test the possible effect of the sample on enzymatic activity, following assay was performed. The cell suspension (2 × 10^6^ cells) in 2 mL of Tyrode’s buffer was sonicated for 5 min. The solution was then centrifuged, and the supernatant was diluted with 8 mL of Tyrode’s buffer. The enzyme solution (45 µL) and test sample solution (5 µL) were transferred into a 96-well microplate and enzyme activity was examined as described above (Section 3.7). All experiments were repeated three times.

### 3.9. Direct Degranulation β-Hexosaminidase Assay Induced by the Sample

The degree of β-hexosaminidase release triggered by the sample in RBL-2H3 cells was determined by a modified β-hexosaminidase release assay. Briefly, RBL-2H3 cells (4 × 10^4^ cells/well) were seeded in a 48-well plate and treated with the samples for 10 h. Tyrode’s buffer supplemented with 5.6 mM glucose, 2 mg/mL BSA and 2 mM glutamine was used to prepare the samples and treat the cells. Then, 50 μL of supernatants were transferred into a 96-well microplate and examined as described above (Section 3.7). A23187 (1 µM) was used as a positive control. All experiments were repeated three times.

### 3.10. Preparation of Human Neutrophils

Human neutrophils from venous blood of healthy, adult volunteers (20–30 years old) were isolated using a standard method of dextran sedimentation prior to centrifugation in a Ficoll-Hypaque gradient and hypotonic lysis of erythrocytes [30]. Purified neutrophils containing >98% viable cells, as determined by the trypan-blue exclusion method [31], were resuspended in a Ca^2+^-free Hank’s buffered salt solution (HBSS) at pH 7.4 and were maintained at 4 °C prior to use.

### 3.11. Superoxide Anion Generation Assay and Elastase Release Inhibition Assay

Neutrophil superoxide anion generation was determined using superoxide dismutase (SOD)-inhibitory cytochrome reduction according to described procedures [32,33]. Degranulation of azurophilic granules was determined by measuring the elastase release as described previously [33]. All experiments were repeated at least three times.

### 3.12. Cytotoxic Assay

MTT assay was used according to the method described before [34]. Briefly, HepG2 (1 × 10^4^ cells), A549 (5 × 10^3^ cells), and MDA-MB-231 (1 × 10^4^ cells) were seeded into 96-well plates, followed by treatment with the AMS samples at concentration of 20 µg/mL. After 72 h, the medium was removed and 100 µL of MTT solution (0.5 mg/mL) was added to each well. The plates were then incubated at 37 °C for 1 h and then, the MTT dye was detected by the addition of DMSO (100 μL). The absorbance was recorded at 550 nm. Doxorubicin was used as a positive control.

### 3.13. Statistics

The results were expressed as mean ± SD unless otherwise specified. The IC_50_ values were calculated using the Microsoft Office (linear function). Statistical significance was calculated by one-way analysis of variance (ANOVA), followed by Dunnett’s test (SigmaPlot, Jandel Scientific, San Rafael, CA, USA). Values with * *p* < 0.05, ** *p* < 0.001 were considered statistically significant.

## 4. Conclusions

The present investigation revealed bioactive fractions and pure principle from the extract of AMS. It resulted in the isolation of the active pure compound, aquimavitalin (**1**). The remarkable inhibitory activity of **1** on mast cell degranulation with nanomolar IC_50_ values provides evidence that phorbol ester could possess antiallergic activity.

Moreover, high potency of phorbol esters may shed light on the use of *A. malaccensis* seeds in the treatment diseases related to allergy. However, further studies are needed to examine the safety of these materials in therapy.

## Figures and Tables

**Table 1:** Antiallergic activity of *Aquilaria malaccensis* seeds extracts, fractions and aquimavitalin (**1**).

Sample	Viability, RBL-2H3	Inhibition of β-Hexosaminidase Release, Degranulation Assay, RBL-2H3 Cells ^a^				Inhibitory Effect on Enzyme
	IC_50_ (μg/mL) ^b^ (% Viability at 100 μg/mL)	A23187-Induced IC_50_ (μg/mL) ^b^	Therapeutical Index ^c^	Antigen-Induced IC_50_ (μg/mL) ^b^	Therapeutical Index ^c^	β-Hexosaminidase (%) ^d^
A-EtOH	>100 (86.0%)	0.92	>109.0	3.9	>25.7	12.7 ± 4.2 (100 μg/mL)
A-BuOH	>100 (93.3%)	1.1	>92.1	6.0	>16.7	7.3 ± 5.5 (100 μg/mL)
A-Water	>100 (94.0%)	–	–	–	–	N/A ^e^
A-EtOAc	>100 (90.3%)	0.56	>177.9	0.86	>116.8	13.3 ± 2.1 (100 μg/mL)
A-Hexane	>100 (95.3%)	0.83	>120.1	5.1	>19.5	13.7 ± 2.5 (100 μg/mL)
A-MeOH	96.8	0.0089	10,910.9	0.069	1405.2	5.3 ± 3.2 (10 μg/mL)
AM4	98.0	0.0034	28,677.6	0.0065	15,098.4	4.7 ± 4.0 (10 μg/mL)
AM4-4	70.6	4.8 × 10^−5^	1,477,328.2	6.8 × 10^−4^	103,776.5	N/A ^e^
AM4-4-7	73.8	7.4 × 10^−4^	99,680.2	0.0065	11,309.9	N/A ^e^
AM4-4-8	73.4	7.6 × 10^−6^	9,645,374.3	8.0 × 10^−5^	917,440.9	N/A ^e^
Aquimavitalin (**1**)	71.5	0.0010 (0.0017 μM)	71,538.5	0.0068 (0.011 μM)	10,550.2	4.3 ± 4.5 (10 μg/mL)

^a^ Dexamethasone (10 nM) inhibited 54.0% ± 4.0% of A23187-induced β-hexosaminidase release and 54.3% ± 7.2% of antigen-induced β-hexosaminidase release; ^b^ IC_50_ values express the concentration of the sample required to inhibit cell growth or degranulation by 50%; ^c^ Therapeutic index was calculated by dividing IC_50_ value from 3-(4,5-dimethylthiazol-2-yl)-2,5-diphenyltetrazolium bromide (MTT) viability assay with corresponding IC_50_ value from degranulation assay; ^d^ Results are presented as mean ± SD (*n* = 3); ^e^ N/A, not applicable; A-EtOH: crude ethanolic extract of *Aquilaria malaccensis* seeds; A-BuOH: n-butanol layer from *Aquilaria malaccensis* seeds; A-Water: water layer from *Aquilaria malaccensis* seeds; A-EtOAc: ethyl acetate layer from *Aquilaria malaccensis* seeds; A-Hexane: n-hexane layer from *Aquilaria malaccensis* seeds; A-MeOH: methanol layer from *Aquilaria malaccensis* seeds, AM: subfractions of methanol layer from *Aquilaria malaccensis* seeds.

**Table 2:** Antiinflammatory effects of *A. malaccensis* seeds extracts on superoxide anion generation and elastase release in fMLP/CB-induced human neutrophils ^a^.

Sample	Superoxide Anion Generation (Inh %)		Elastase Release (Inh %)	
A-EtOH	90.1 ± 5.3	**	85.3 ± 0.8	**
A-BuOH	93.9 ± 8.3	**	77.6 ± 2.4	**
A-Water	11.4 ± 1.6	*	2.7 ± 4.1	–
A-EtOAc	94.8 ± 5.6	**	85.4 ± 1.8	**
A-Hexane	103.4 ± 1.8	**	80.2 ± 4.0	**
A-MeOH	96.5 ± 8.0	**	90.4 ± 6.0	**
AM1	54.5 ± 5.7	**	99.2 ± 2.3	**
AM2	68.7 ± 5.0	**	47.5 ± 5.3	**
AM3	105.9 ± 3.4	**	86.8 ± 2.0	**
AM4	100.7 ± 8.1	**	70.9 ± 1.0	**
AM5	102.6 ± 1.5	**	93.5 ± 3.7	**
AM6	102.4 ± 2.0	**	99.3 ± 2.3	**

^a^ Percentage of inhibition (Inh %) at 10 µg/mL concentration; results are presented as mean ± SEM (*n* = 3–4); * *p* < 0.05, ** *p* < 0.001 compared with the control value (formyl-methionyl-leucyl-phenylalanine/cytochalasin B, fMLP/CB).

**Table 3:** Cytotoxic screening of *A. malaccensis* seeds extracts on cancer cell lines ^a^.

Sample	HepG2 ^b^	MDA-MB231 ^c^	A549 ^d^
A-EtOH	16.0	37.2	29.7
A-BuOH	4.2	34.4	57.1
A-Water	−9.3	6.8	13.3
A-EtOAc	1.5	41.2	23.5
A-Hexane	25.1	42.5	16.8
A-MeOH	−0.8	30.3	32.7
AM1	8.1	1.7	−12.6
AM2	2.4	11.5	19.8
AM3	25.5	46.3	39.5
AM4	23.4	56.5	79.3
AM5	7.9	39.9	29.2
AM6	5.3	56.0	39.5
doxorubicin ^e^	91.3	97.7	98.0

^a^ Percentage of inhibition (%) at 20 µg/mL concentration (*n* = 1); ^b^ Hep-G2: human hepatocellular carcinoma cells; ^c^ MDA-MB231: human breast adenocarcinoma cells; ^d^ A549: human lung adenocarcinoma cells; ^e^ Positive control (2 µg/mL).

**Table 4:** 1D and 2D NMR data of aquimavitalin (**1**) in CDCl_3_^a^.

Position	*δ*_H_, Multiplicity (*J* in Hz)	*δ*_C_, Type	COSY (^1^H–^1^H)	HMBC (^1^H–^13^C)	NOESY (^1^H–^1^H)
1	7.57 (s)	160.8 CH	10, 19	4, 10	18
2	–	132.8 C	–	–	–
3	–	209.3 C	–	–	–
4	–	73.6 C	–	–	–
5*α*	2.48 (d, *J* = 18.8)	38.3 CH_2_	7	4, 6, 7	5, 20
5*β*	2.58 (d, *J* = 18.8)	–	–	–	–
6	–	140.6 C	–	–	–
7	5.68 (brs)	129.1 CH	5, 8	14, 20	14, 20
8	3.26 (t, *J* = 5.2)	38.8 CH	7, 14	6, 14, 15	11, 17
9	–	78.4 C	–	–	–
10	3.22 (brs)	55.9 CH	1, 19	–	–
11	2.13 (m)	43.0 CH	12, 18	–	17, 18
12	5.43 (d, *J* = 10.4)	75.9 CH	11	11, 13, 15, 18, 1′	18
13	–	65.7 C	–	–	–
14	1.08 (d, *J* = 5.2)	36.1 CH	8	7, 13, 15, 16	–
15	–	25.6 C	–	–	–
16	1.19 (s)	23.8 CH_3_	–	13, 14, 15, 17	–
17	1.24 (s)	16.7 CH_3_	–	13, 14, 15, 16	–
18	0.88 (d, overlap)	14.0 CH_3_	11	9, 11, 12	–
19	1.73 (brs)	10.0 CH_3_	1, 10	1, 2, 3	–
20a	4.02 (d, *J* = 12.8)	67.9 CH_2_	–	5, 6, 7	–
20b	3.95 (d, *J* = 12.8)	–	–	–	–
21	–	173.9 C	–	22	–
22	2.10 (s)	21.1 CH_3_	–	–	–
1′	–	166.3 C	–	–	–
2′	5.57 (d, *J* = 11.2)	115.6 CH	3′	–	3′
3′	6.59 (t, *J* = 11.6)	145.6 CH	2′, 4′	1′, 5′	–
4′	7.39 (dd, *J* = 15.2 and 11.6)	126.5 CH	3′, 5′	–	6′
5′	6.46 (dd, *J* = 14.8 and 10.4)	142.4 CH	4′, 6′	–	7′
6′	6.20 (dd, *J* = 15.2 and 10.8)	130.1 CH	5′, 7′	–	–
7′	5.92 (dt, *J* = 15.2 and 7.2)	141.0 CH	6′, 8′	–	–
8′	2.13 (m)	33.0 CH_2_	7′, 9′	6′, 7′, 9′	9′
9′	1.38 (m)	28.9 CH_2_	8′	10′	–
10′	1.26–1.28 (m, overlap)	29.1 CH_2_	–	–	–
11′	1.26–1.28 (m, overlap)	29.1 CH_2_	–	–	–
12′	1.26–1.28 (m, overlap)	31.7 CH_2_	–	–	–
13′	1.26–1.28 (m, overlap)	22.6 CH_2_	–	–	–
14′	0.86 (t, *J* = 7.2)	14.4 CH_3_	–	12′, 13′	–

**^a^** ^1^H and ^13^C NMR data (*δ*) were measured at 400 and 100 MHz, respectively; chemical shifts are in ppm; COSY: Correlation spectroscopy; HMBC: Heteronuclear multiple bond correlation spectroscopy; NOESY: Nuclear Overhauser effect spectroscopy.

## Supplementary Materials

Supplementary materials can be found at http://www.mdpi.com/1422-0067/17/3/398/s1.

## Abbreviations

AMS: *Aquilaria malaccensis* seeds
A-EtOH: Crude ethanolic extract of *Aquilaria malaccensis* seeds
A-BuOH: *n*-Butanol layer from *Aquilaria malaccensis* seeds
A-Water: Water layer from *Aquilaria malaccensis* seeds
A-EtOAc: Ethyl acetate layer from *Aquilaria malaccensis* seeds
A-Hexane: *n*-Hexane layer from *Aquilaria malaccensis* seeds
A-MeOH: Methanol layer from *Aquilaria malaccensis* seeds
AM: Subfractions of methanol layer from *Aquilaria malaccensis* seeds
RBL-2H3: Rat basophilic leukemia cells
HepG2: Human hepatocellular carcinoma cells
A549: Human breast adenocarcinoma cells
MDA-MB231: Human lung adenocarcinoma cells
fMLP/CB: Formyl-methionyl-leucyl-phenylalanine/cytochalasin B
