# Supplementary material for: Antiallergic Phorbol Ester from the Seeds of Aquilaria malaccensis
Source: Int J Mol Sci. 2016 Mar 21;17(3):398. doi: 10.3390/ijms17030398 (PMC4813253; doi:10.3390/ijms17030398)
Supplement: Supplementary file 1 [file ijms-17-00398-s001.zip › ijms-117707-Supplementary Materials/ijms-117707-supplementary-Figure S1-S8.pdf]

Supplementary Materials: Antiallergic Phorbol Ester from the Seeds of Aquilaria Malaccensis

Michal Korinek, Vitthal D. Wagh, I-Wen Lo, Yu-Ming Hsu, Hsue-Yin Hsu, Tsong-Long Hwang, Yang-Chang Wu, Yuan-Bin Cheng, Bing-Hung Chen and Fang-Rong Chang

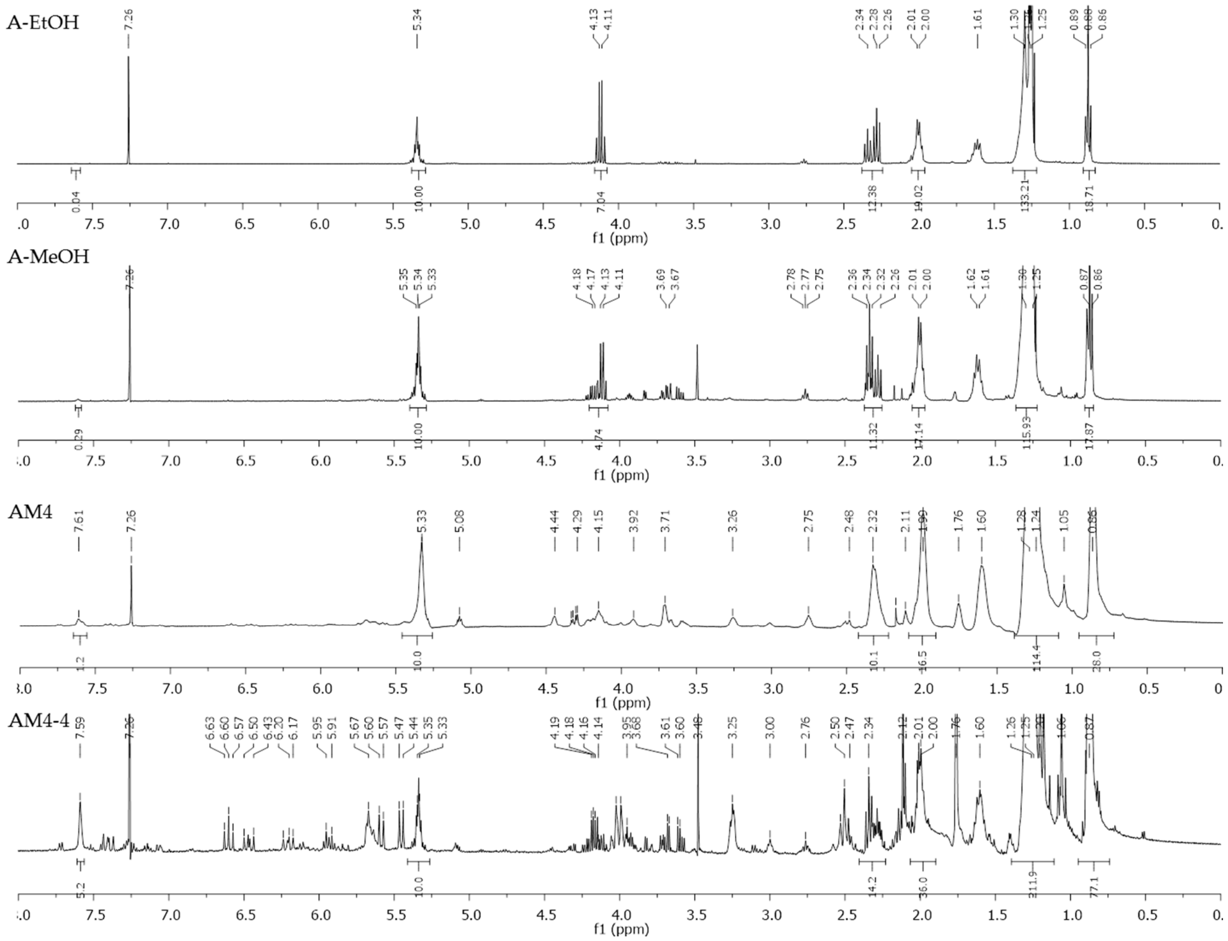

Figure S1. ^{1}H NMR spectra of A-EtOH, A-MeOH, AM4 and AM4-4 in $CDCl_3$, 400 MHz. The ^{1}H NMR comparison of consequent fractions guided by antiallergic activity. The typical signals for phorbol diterpenes (δ_H 7.5, H-1; δ_H 5.6, H-7) increased with purification in following manner: ethanolic extract (A-EtOH) < methanolic partition layer (A-MeOH) < 4th fraction of the methanolic layer (AM4) < 4th fraction of AM4 (AM4-4).

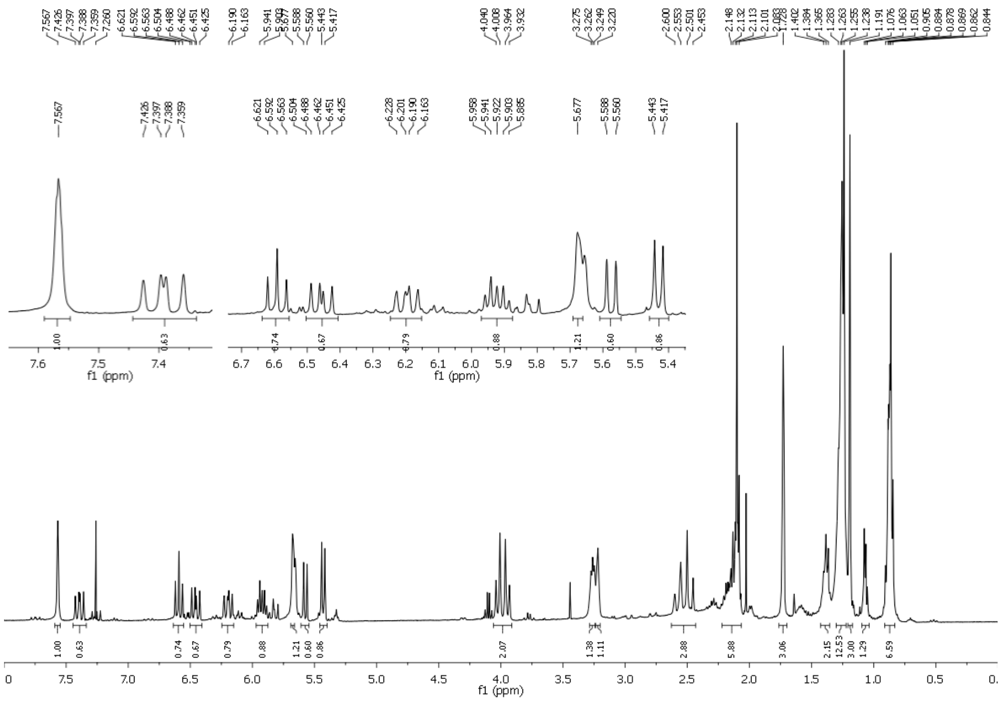

Figure S2. The ^{1}H NMR spectrum of aquimavitalin (**1**) in $CDCl_3$, 400 MHz.

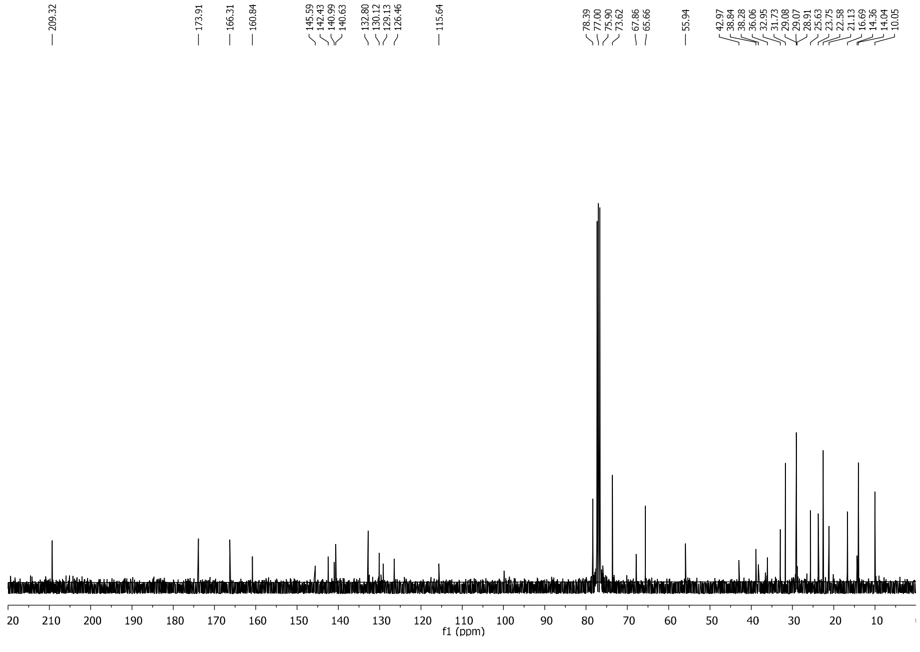

Figure S3. The ^{13}C NMR spectrum of aquimavitalin (**1**) in $CDCl_3$, 100 MHz.

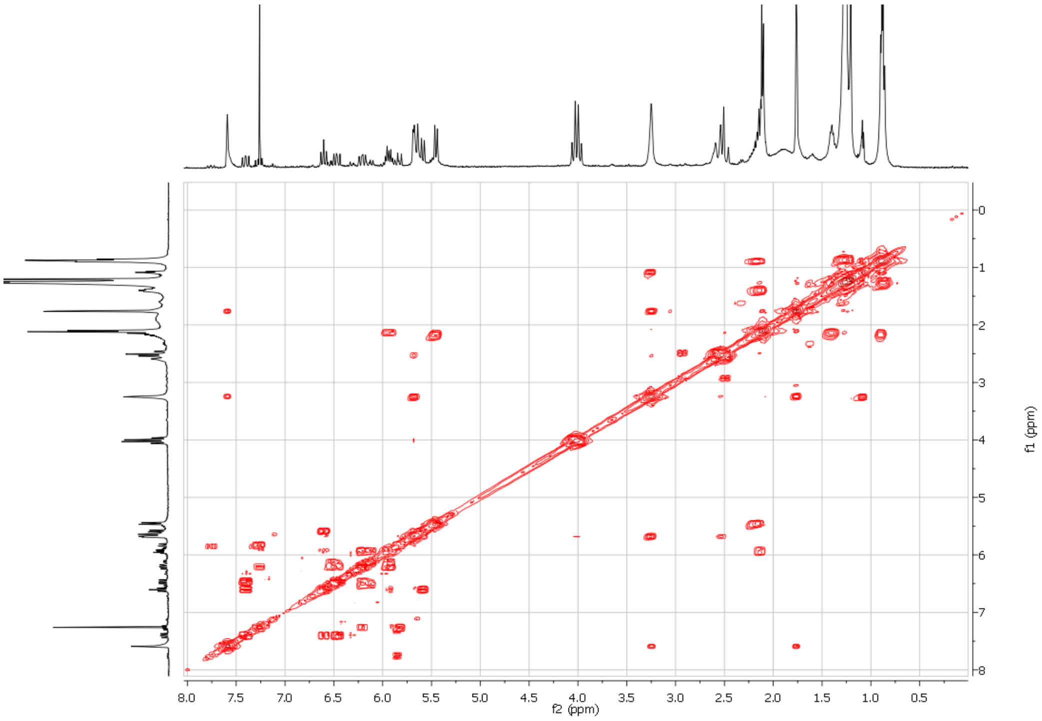

Figure S4. ^1H–^1H correlation spectroscopy (COSY) spectrum of aquimavitalin (**1**) in $CDCl_3$.

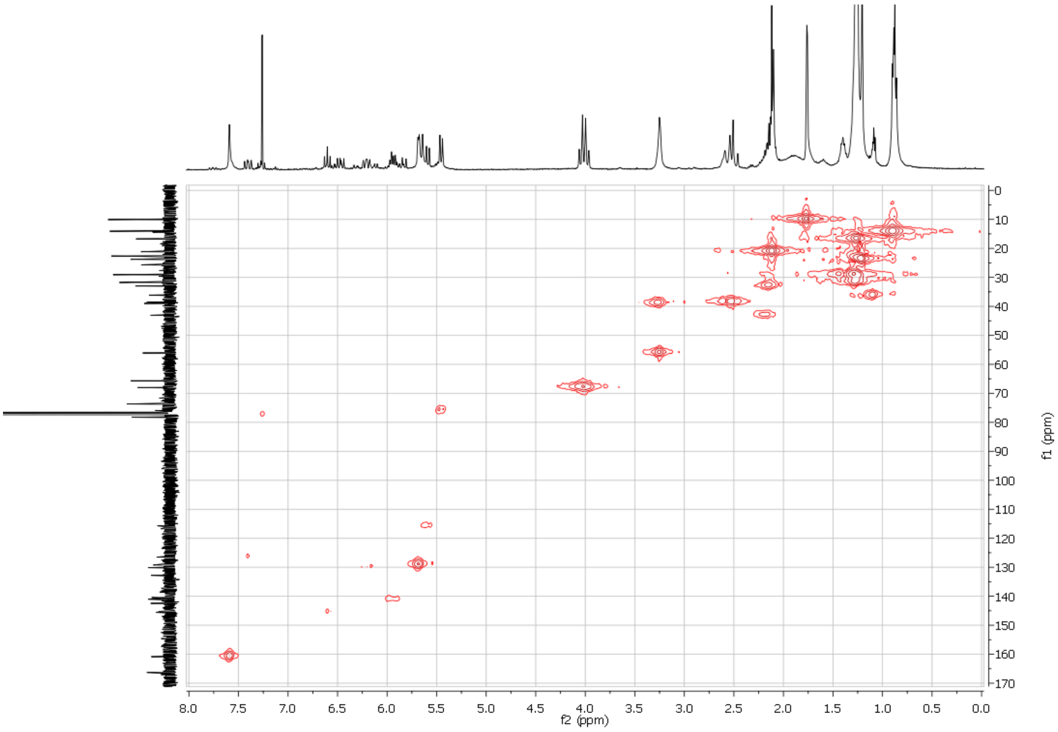

Figure S5. HMQC spectrum of aquimavitalin (**1**) in $CDCl_3$.

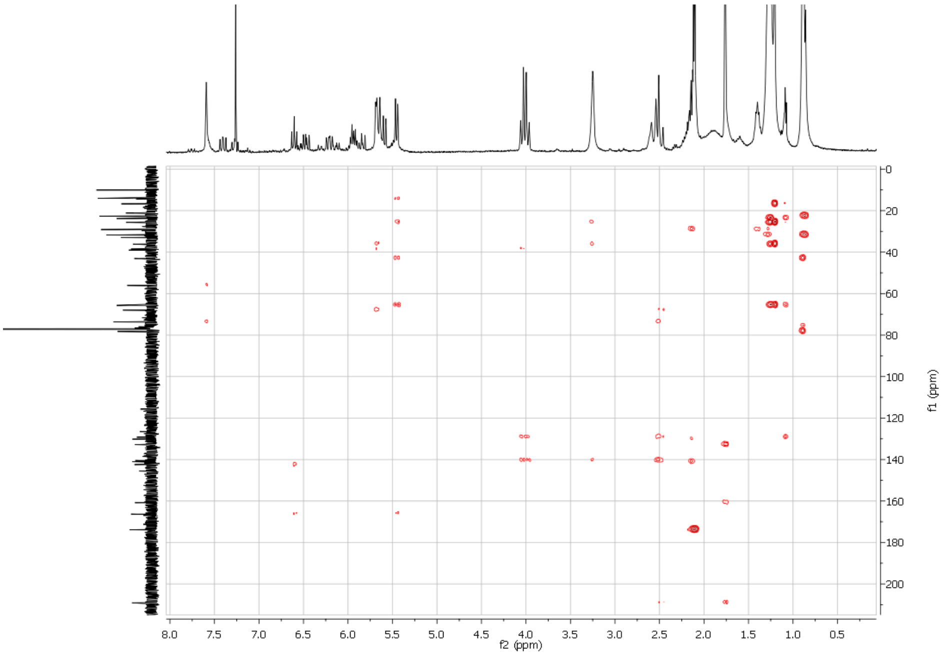

Figure S6. Heteronuclear multiple bond correlation spectroscopy (HMBC) spectrum of aquimavitalin (**1**) in $CDCl_3$.

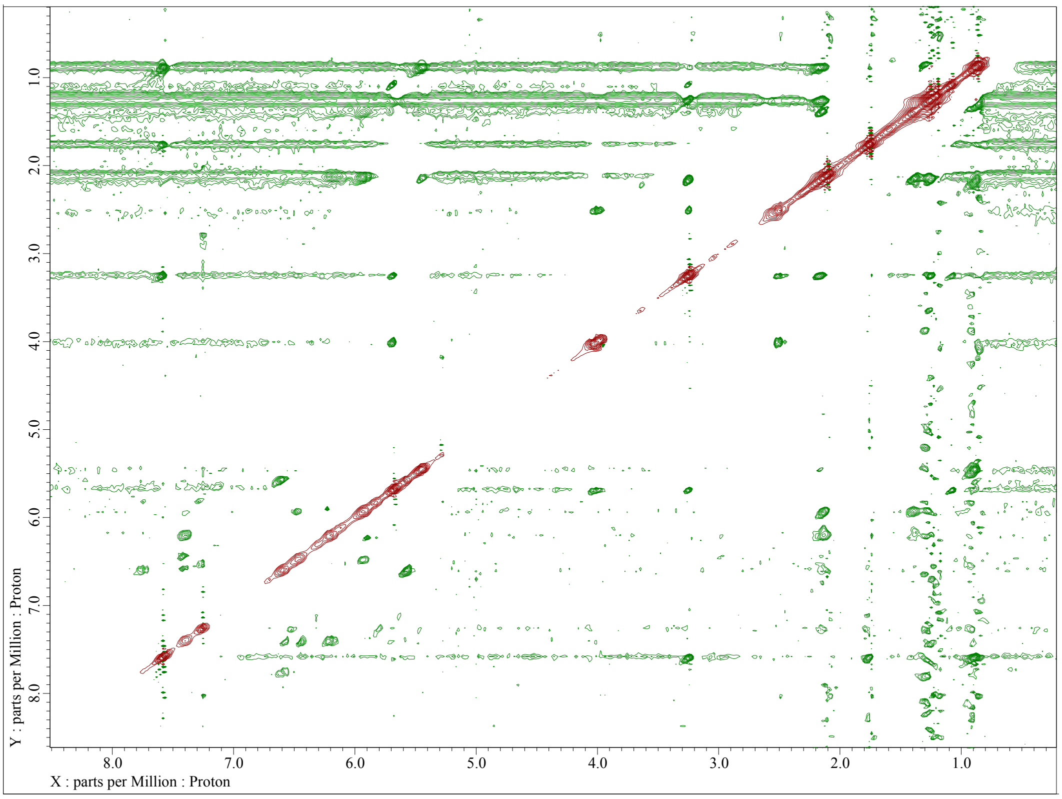

Figure S7. Nuclear overhauser effect spectroscopy (NOESY) spectrum of aquimavitalin (**1**) in $CDCl_3$.

Mass Spectrum SmartFormula Report

Analysis Info

Analysis Name	D:\Data\c4\am449_000005.d	11/5/2015 12:08:53 PM
Method	broadband first signal	Operator: YU HSIAO-CHING
Sample Name	am4-4-9	Instrument: BRUKER FT-MS solariX
Comment	ESI Positive	

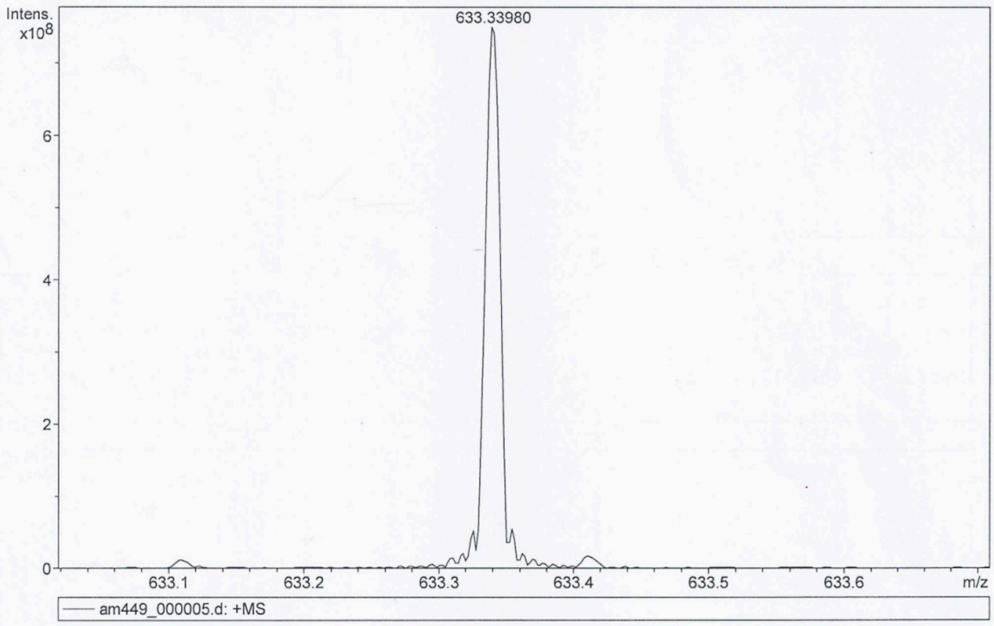

Meas. m/z	#	Formula	Score	m/z	err [mDa]	err [ppm]	mSigma	rdb	e⁻ Conf	N-Rule
633.33980	1	C 36 H 50 Na O 8	100.00	633.33979	-0.01	-0.01	12.3	11.5	even	ok

Figure S8. High-resolution electrospray ionization mass spectrometry (HR-ESIMS) spectrum of aquimavitalin (**1**).
